# Case Report: Infection With SARS-CoV-2 in the Presence of High Levels of Vaccine-Induced Neutralizing Antibody Responses

**DOI:** 10.3389/fmed.2021.704719

**Published:** 2021-07-23

**Authors:** Bianca Schulte, Benjamin Marx, Marek Korencak, Dorian Emmert, Souhaib Aldabbagh, Anna Maria Eis-Hübinger, Hendrik Streeck

**Affiliations:** Institute for Virology, University Hospital Bonn, Bonn, Germany

**Keywords:** SARS-CoV-2, breakthrough infection, mRNA vaccines, virus variants, neutralizing antibodies

## Abstract

We present a case of SARS-CoV-2 B.1. 525 infection in a healthcare worker despite the presence of highly neutralizing, multivariant-specific antibodies 7 weeks after full vaccination with the mRNA vaccine BNT162b2. We show that the virus replicated to high levels in the upper respiratory tract over the course of several days in the presence of strong antibody responses. The virus was readily propagatable *in vitro*, demonstrating the potential to transmit to others, bolstered by the fact that several household members were equally infected. This highlights the importance of protective measures even in vaccinated individuals.

## Introduction

The vaccines available against SARS-CoV-2 have shown great efficacies but are not in all cases protective (1–3). Thus, infections after vaccinations can be expected in a few individuals. In particular with emerging SARS-CoV-2 variants more breakthrough infections are expected. However, studies have shown that vaccinated individuals have lower viral shedding (4) and a shorter duration of SARS-CoV-2 infection. Here we report on a case of SARS-CoV-2 infection with high levels of pharyngeal viral shedding 7 weeks after full vaccination with BNT162b2, despite the presence of high titers of anti-spike-antibodies with good neutralizing activity against the original SARS-CoV-2 variant and the contemporaneous variant B.1.525.

## Case Description

### Longitudinal Follow-Up of Breakthrough SARS-CoV-2 Infection

**Figure 1:**
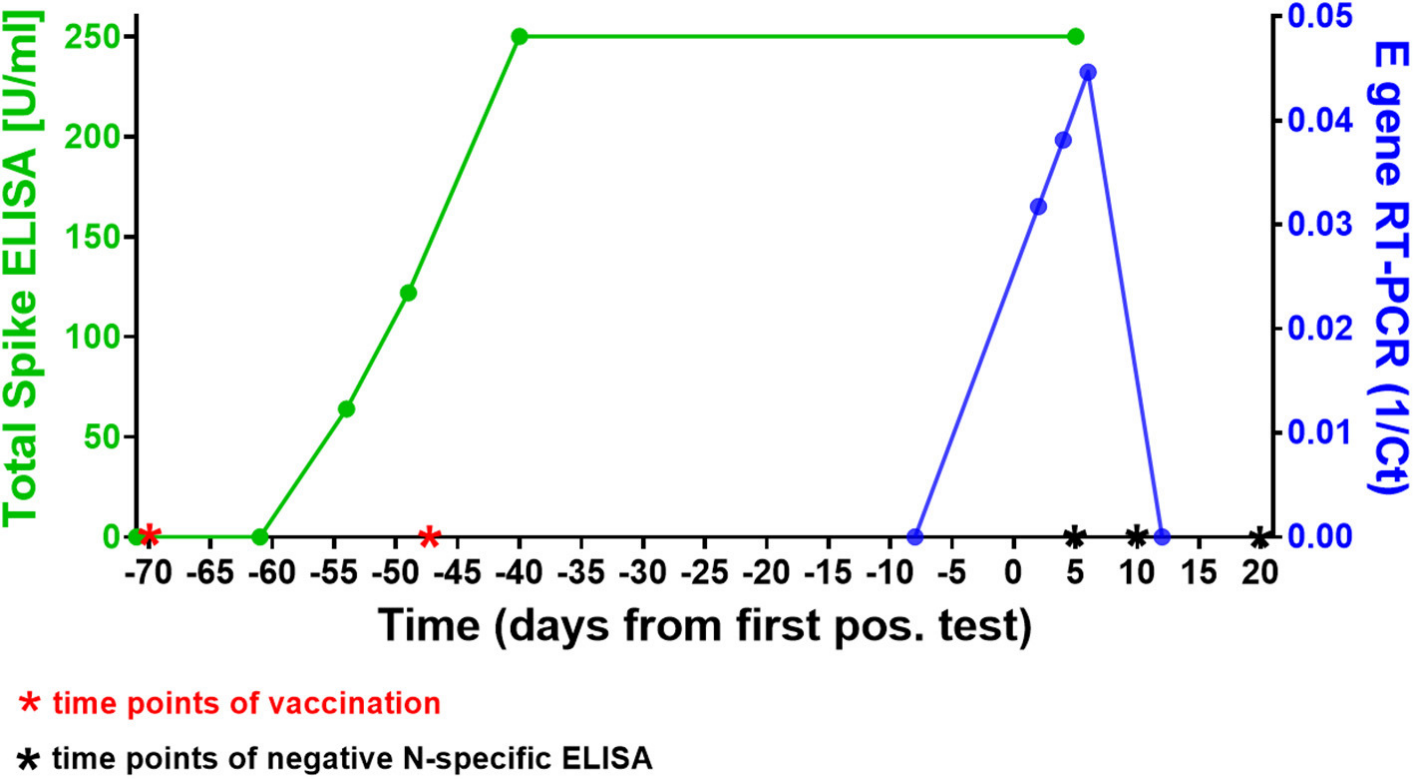
Time course of SARS-CoV-2 vaccinations, antibody response, and infection in our subject. The green line shows the course of SARS-CoV-2 spike-specific antibodies in the patient. A first rapid antigen test was positive 49 days after the second vaccination, a first positive RT-PCR 2 days later. Note that 250 U/ml were the upper threshold value of the ELISA, the result for both data points in this assay was above the limit of accurate detection (>250 U/ml). The blue line shows the SARS-CoV-2 RT-PCR cycle threshold (Ct) value of the laryngopharyngeal swab samples taken from the patient on 5 different days around the time point of infection (results shown here for E gene Ct's). Note that the first positive RT-PCR showed the lowest viral load (highest Ct) of all three positive tests. The Roche Elecsys® N-specifc ELISAs performed on days 5, 10, and 20 days after first positive SARS-CoV-2 antigen test were negative at COIs 0.065, 0.46, and 0.569, respectively (threshold for positive result: 1.0), see also Supplementary Figure 1.

Forty-nine days after receiving his second dose of SARS-CoV-2 mRNA vaccine BNT162b2 a 42-year old, otherwise healthy male tested positive for SARS-CoV-2 in a rapid antigen test, which was confirmed in a laryngopharyngeal swab by RT-PCR for two viral targets (E and S genes as previously described), two days later (see Figure 1). All four members of the household equally tested positive for SARS-CoV-2 (the two children 9 days before the individual whose case is presented here, and the wife 10 days after that) and all individuals had asymptomatic infections, except for the wife who reported one mild symptom (loss of sense of taste). At the time of her first positive test she had been vaccinated once with ChAdOx1-S (AstraZeneca) 3 weeks prior. Subsequent sequential testing of the male subject for SARS-CoV-2 RNA demonstrated an increase in viral load with a peak at day 6 after the first positive SARS-CoV-2 rapid antigen test. The first negative test was confirmed on day 10. Virus Propagation showed high titers after 5 day culture on Caco-2 cells (E gene RT-PCR *Ct* = 9.44, S gene *Ct* = 14.53) and capable productively infecting and lysing 100% of VeroE6 cells in 3 days (E gene *Ct* = 14.28, S gene *Ct* = 13.23). NextGen Sequencing using the Qiagen protocol on a miSeq (Illumina) platform (5) identified the virus from the laropharyngeal swab as SARS-CoV-2 B.1.525, identical to the reference sequence EPI_ISL_1571188, except for three silent base substitutions in the spike gene (A23746G, T24070C, and G25767T). This variant had been detected at varying but low frequencies in the German population in recent months, but remained a minor variant under investigation (6).

### Good Neutralization Capacity Despite High Viral Titers

**Figure 2:**
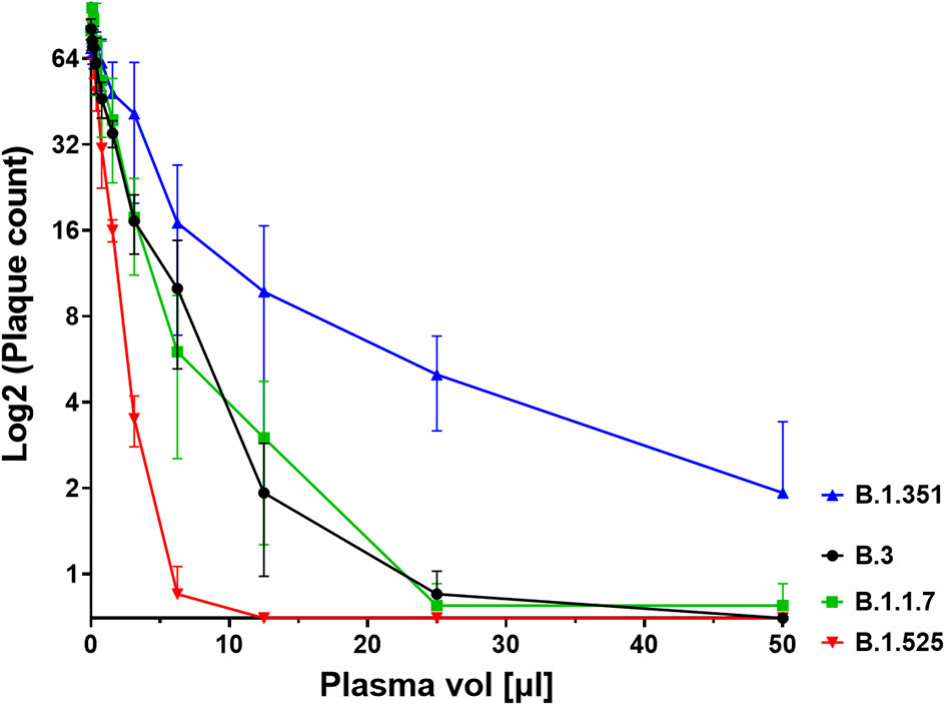
Plaque count after neutralisation assay with serial dilutions of the patient's serum on SARS-CoV-2 variants B.3, B.1.1.7, B.1.351, and B.1.525. The capacity of the serum to neutralise the different variants was correlated to the prevention of plaque formation. The plasma sample was diluted between a range of 1:4 (50 μl in 200 μl, see x axis) and 1:2048 to assess its titer for neutralisation. Results of four independent experiments are shown. Variations in NT values between experiments are indicated by an error bar.

**Figure 3:**
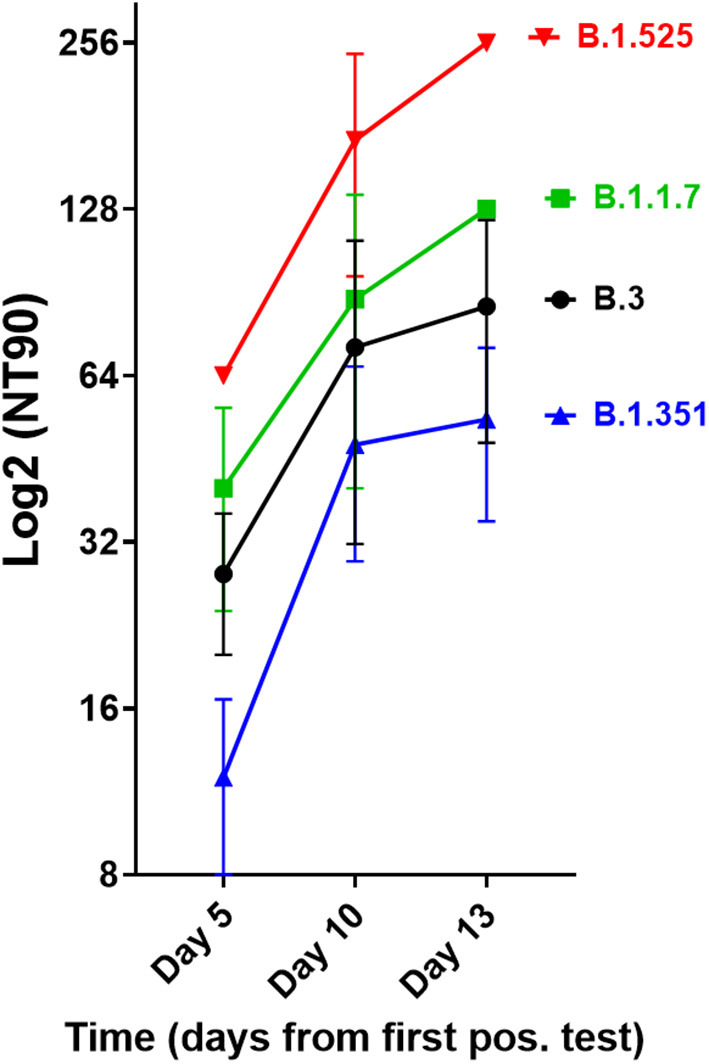
Neutralisation capacity of patient's plasma on days 5, 10, and 13 after first positive SARS-CoV-2 result. Neutralisation assays were performed with the four virus variants B.3, B.1.1.7, B.1.351, and B.1.525, the latter of which the patient had been infected with. The NT90 value is the corresponding reciprocal of the dilution at which 90% of plaques were suppressed. Results of three independent experiments are shown. Variations in NT values between experiments are indicated by an error bar.

We next investigated the ability of SARS-CoV-2 recognition by the vaccine-induced immune responses. Using the Roche whole antibody ELISA (spike subdomain S1-specific) we first detected anti-SARS-CoV-2 antibodies 16 days after the first vaccination. Maximum levels were reached 9 days after the second vaccination (Figure 1). To determine the patient's ability to recognize and neutralize the SARS-CoV-2 variant B.1.525 as well as other variants we performed plaque reduction neutralization assays as previously described (7). We longitudinally tested plasma from the patient in the neutralization assays against four major SARS-CoV-2 variants B.3, B.1.117 (α variant), B.1.351 (β), and B.1.525 (η). Surprisingly, the plasma was equally capable of neutralizing B.1.525 virus as the other variants with slightly lower NT activity against B.1.351 (see Figure 2). Overall we observed a general trend for an increase in neutralization between day 5 and 13 after SARS-CoV-2 breakthrough infection (Figure 3). This boost detected in neutralization capacities of the individual's plasma from day 5 to day 13 was similar irrespective of the virus tested, i.e., at least a doubling of the NT90 values was observed against all four virus variants. Surprisingly, in these neutralizing samples no nucleocapsid (N)-specific antibodies could be detected up to 20 days after infection (Figure 1, Roche Elecsys® Anti-SARS-CoV-2 immunoassay and Supplementary Figure 1), while spike (S)-specific antibodies stayed at the highest detectable levels or above (Euroimmun SARS-CoV-2 ELISA).

## Discussion

Although high vaccination effectiveness (>90%) has been shown for several COVID-19 vaccines (1–3), questions remain as to the composition and characteristics of the group of individuals developing breakthrough infections despite full vaccination. It is believed that breakthrough infection occur due to immune escape mutations or lack of presence of high titers of neutralizing antibody responses. Here we describe a case of an individual that became infected despite the presence of high titer neutralizing antibodies. He developed high levels of viral load detectable in the pharyngeal swabs, and was likely to infect others. All four members in the familiy were infected, but we can not conclusively show who infected whom. However, the case raises the question whether breakthrough infections with SARS-CoV-2 usually occur in spite of strong neutralizing antibody responses. Indeed, while most reported cases of breakthrough infection ocurred before full vaccine-induced immunity had been induced (8), rare breakthrough infection cases were described despite the presence of neutralizing capacities against the infecting variant (9). Interestingly, this case and other breakthrough infection cases we investigated (unpublished data) show that the absence of an N-specific antibody response after infection is common. This led us to hypothesize that highly efficient spike-based neutralization in vaccinees prevents mounting antibody responses to nucleocapsid, an otherwise highly immunogenic viral protein (10).

There are many possible causes for breakthrough infection in adults. Firstly, immunosenescence might play a role in that the aging immune system is known to produce lower numbers of naive T and B cells (11). Moreover, reduced vaccination efficiency is usually observed above the age of 65 (12), yet our described case was 42 years old. Secondly, the longevity of specific B cells might differ between individuals, but the individual mounted high titers of neutralizing antibodies. While he became infected with the B.1.525 variant, he developed antibodies after BNT162b2 vaccination that efficiently neutralized the SARS-CoV-2 strains B.3 and B.1.1.7 as well as B.1.525. When comparing the neutralization capacities against the variants we determined that they differed by a factor of up to 4, with B.1.525 being neutralized the best, followed by B.3 and B.1.1.7, and B.1.351 being neutralized to the least degree among the four. This indicates that the amino acid differences in spike between these variants are still crucial for the efficiency of neutralizing antibodies even after natural infection of vaccinees. Whether B.1.525 is more efficiently neutralized by vaccination-induced antibodies than variants of concern or the early variant B.3 remains to be shown in larger studies. However, a first small study of BNT162b2 vaccinees (*n* = 37) found significantly higher neutralization of B.1.525 and B.1.1.7 and weaker neutralization of B.1.351 compared to B.1, respectively, thus corroborating our results (13). The slightly different result with B.1.1.7 might be due to the fact that we compared its neutralization to B.3, a variant more dominant in Germany.

Because of the scarcity of breakthrough infections it has been very difficult to fully characterize this phenomenon via systematic longitudinal studies. For COVID-19, very few cases of breakthrough infection have been reported and conclusive documentation of fully established immunity before infection is widely lacking (9). Therefore, based on the case presented here we propose that there might be another cause for breakthrough infection with SARS-CoV-2 than the commonly described ones discussed above.

## Patient Perspective and Conclusion

This case illustrates one example of SARS-CoV-2 infection despite the presence of highly neutralizing, multivariant-specific antibodies after full vaccination with the mRNA vaccine BNT162b2. Furthermore, it demonstrates that the virus can replicate to high levels in the upper respiratory tract over the course of several days in the presence of strong antibody responses. Especially the high viral loads measured in RT-PCR and the fact that the virus from the patient was propagatable *in vitro* make it likely that some fully vaccinated individuals can transmit the virus to others highlighting the importance of protective measures even of vaccinated individuals.

## Data Availability Statement

The raw data supporting the conclusions of this article will be made available by the authors, without undue reservation.

## Ethics Statement

Ethical review and approval was not required for the study on human participants in accordance with the local legislation and institutional requirements. The patients/participants provided their written informed consent to participate in this study.

## Author Contributions

BM and BS: conceptualization, software, investigation, data curation, and project administration. BS, BM, DE, MK, and SA: methodology. BM, DE, SA, MK, and BS: validation. BM, BS, DE, and MK: formal analysis. HS and AE-H: resources. BS: writing the original draft preparation and visualization. BS, BM, HS, and AE-H: writing the review and editing. All authors have read and agreed to the published version of the manuscript.

## Conflict of Interest

The authors declare that the research was conducted in the absence of any commercial or financial relationships that could be construed as a potential conflict of interest.

## Publisher's Note

All claims expressed in this article are solely those of the authors and do not necessarily represent those of their affiliated organizations, or those of the publisher, the editors and the reviewers. Any product that may be evaluated in this article, or claim that may be made by its manufacturer, is not guaranteed or endorsed by the publisher.

## Supplementary Material

Supplementary Figure 1 Spike (S) and nucleocapsid (N)-specific ELISAs for SARS-CoV-2 antibodies were performed at 3 different time points after infection. The result of the Euroimmun ELISAs is given as a ratio of extinction of sample/extinction of calibrator and is considered positive between 1.1 and 12. The Roche Elecsys® N ELISA result is given in COI (cutoff index), which is positive between 1.0 and approximately 250.
