# Supplementary figures and images for: Case Report: Infection With SARS-CoV-2 in the Presence of High Levels of Vaccine-Induced Neutralizing Antibody Responses
Source: Front Med (Lausanne). 2021 Jul 23;8:704719. doi: 10.3389/fmed.2021.704719 (PMC8342944; doi:10.3389/fmed.2021.704719)

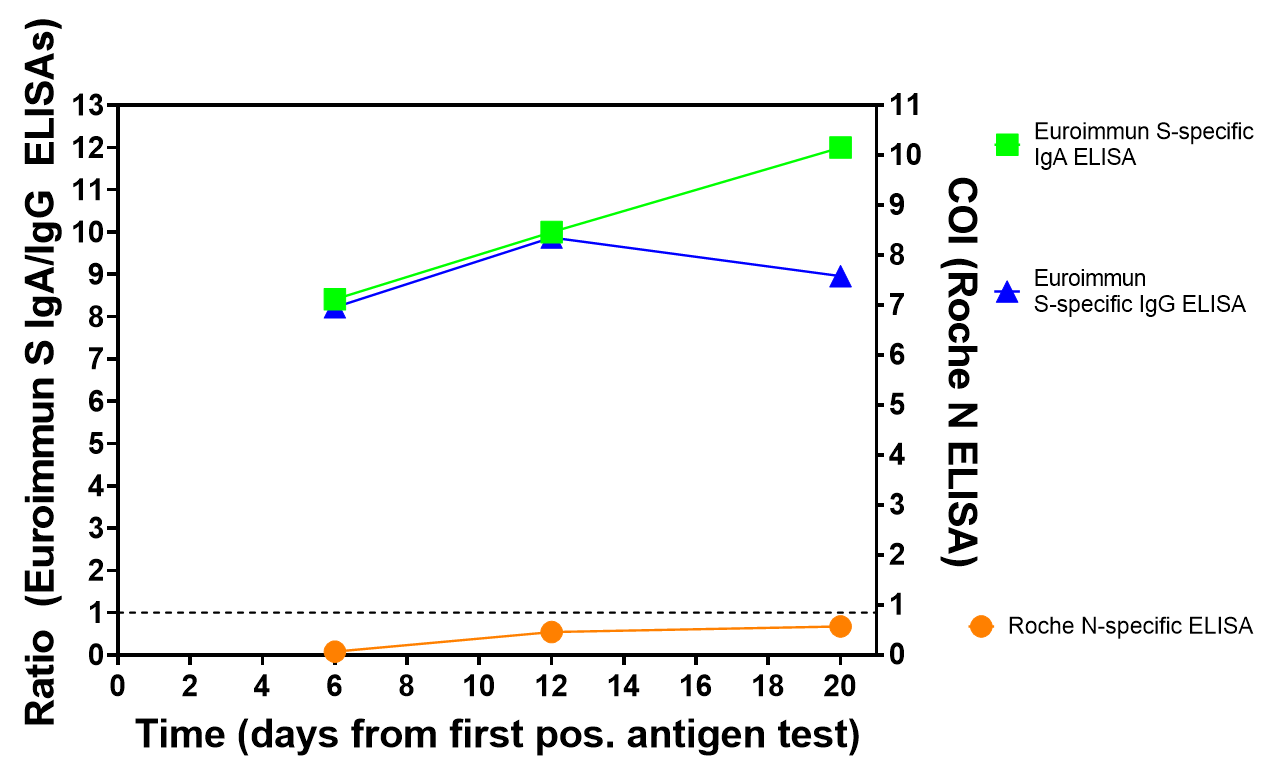

Supplement: Supplementary Figure 1 — Spike (S) and nucleocapsid (N)-specific ELISAs for SARS-CoV-2 antibodies were performed at 3 different time points after infection. The result of the Euroimmun ELISAs is given as a ratio of extinction of sample/extinction of calibrator and is considered positive between 1.1 and 12. The Roche Elecsys® N ELISA result is given in COI (cutoff index), which is positive between 1.0 and approximately 250. [file Image_1.TIF]
